# Sex differences and dietary patterns in the association of air pollutants and hypertension

**DOI:** 10.1186/s12889-024-18620-9

**Published:** 2024-04-23

**Authors:** Chen Zhang, Yuanyuan Wang, Wei Xie, Jingxian Zhang, Ting Tian, Qianrang Zhu, Xinyu Fang, Jing Sui, Da Pan, Hui Xia, Shaokang Wang, Guiju Sun, Yue Dai

**Affiliations:** 1 Key Laboratory of Environmental Medicine and Engineering of Ministry of Education, Department of Nutrition and Food Hygiene, School of Public Health, Southeast University, 210009 Nanjing, China; 2 Institute of Food Safety and Assessment, Jiangsu Provincial Center for Disease Control and Prevention, 210009 Nanjing, China; 3 Research Institute for Environment and Health, Nanjing University of Information Science and Technology, 211544 Nanjing, China

**Keywords:** PM_2.5_, O_3_, Dietary pattern, Sex differences, Hypertension

## Abstract

**Background:**

Hypertension is one of the major public health problems in China. Limited evidence exists regarding sex differences in the association between hypertension and air pollutants, as well as the impact of dietary factors on the relationship between air pollutants and hypertension. The aim of this study was to investigate the sex-specific effects of dietary patterns on the association between fine particulate matter (PM_2.5_), ozone(O_3_) and hypertension in adults residing in Jiangsu Province of China.

**Methods:**

A total of 3189 adults from the 2015 China Adult Chronic Disease and Nutrition Surveillance in Jiangsu Province were included in this study. PM_2.5_ and O_3_ concentrations were estimated using satellite space-time models and assigned to each participant. Dietary patterns were determined by reduced rank regression (RRR), and multivariate logistic regression was used to assess the associations of the obtained dietary patterns with air pollutants and hypertension risk.

**Results:**

After adjusting for confounding variables, we found that males were more sensitive to long-term exposure to PM_2.5_ (Odds ratio (OR) = 1.42 95%CI:1.08,1.87), and females were more sensitive to long-term exposure to O_3_ (OR = 1.61 95%CI:1.15,2.23). Traditional southern pattern identified through RRR exhibited a protective effect against hypertension in males (OR = 0.73 95%CI: 0.56,1.00). The results of the interaction between dietary pattern score and PM_2.5_ revealed that adherence to traditional southern pattern was significantly associated with a decreased risk of hypertension in males (*P* < 0.05), while no significant association was observed among females.

**Conclusions:**

Our findings suggested that sex differences existed in the association between dietary patterns, air pollutants and hypertension. Furthermore, we found that adherence to traditional southern pattern may mitigate the risk of long-term PM_2.5_ exposure-induced hypertension in males.

## Introduction

Hypertension is a major risk factor and component of cardiovascular diseases. In recent years, due to economic development and shifts in societal lifestyle and dietary structure, the global prevalence of hypertension doubled from 1990 to 2019 [1]. Study has shown that 31.1% of the global adult population had hypertension in 2010, and the prevalence of hypertension has been steadily increasing in middle and low-income countries [2]. The results of the 2017 China Disease Burden Study showed that high systolic blood pressure (SBP) was the leading cause of disability adjusted life years (DALYs) loss. It caused 2.54 million deaths in China, of which 95.7% died from cardiovascular disease, bringing a huge economic burden to Chinese healthcare industry [3]. However, hypertension is considered to be the leading preventable cause of premature death and disability worldwide. Therefore, it is critical to find acceptable, population-based and cost-effective strategies to improve hypertension in the population [2, 4].

Particulate matter is a complex mixture of particles with different sizes and composition suspended in the air, which can be formed by human activities and nature, primarily including industrial emissions, vehicular exhausts, waste incineration, wildfires, and sandstorms [5]. Fine particulate matter (PM_2.5_) is a fine particle pollutant with an aerodynamic diameter of ≤ 2.5 μm, related studies have shown that long-term exposure to particulate matter can impair the body’s immune mechanism and destroy cellular immunity [6]. At the same time, the concentration of Ozone (O_3_) in the environment has been increasing in recent years, and the number of deaths due to O_3_ pollution in China reached one-fourth of the world’s deaths due to O_3_ pollution in 2017 [7]. Previous studies have investigated the association between long-term exposure to PM_2.5_ and O_3_ and hypertension prevalence, but the results were inconsistent. Little attention was paid to sex-specific differences between pollutants and hypertension.

Some experimental studies have shown that the increase of cardiovascular disease burden caused by air pollutants is related to oxidative stress and systemic inflammation [8]. It is hypothesized that dietary interventions and antioxidant supplementation may mitigate the adverse effects of air pollutants. Results of a recent UK Biobank analysis demonstrated that adherence to a healthy diet reduced the risk of mortality associated with ambient air pollutants, indicating that individuals with higher vegetable intake exhibited a weakened association between air pollutants and increased all-cause death risk [9], and a prospective study from the US showed that the Mediterranean diet modified the association between PM_2.5_ and cardiovascular disease [10]. However, limited empirical evidence exists regarding the association between dietary patterns and air pollutants in hypertension among the Chinese population. The only two studies were the Dietary Approaches to Stop Hypertension (DASH) diet and the Mediterranean diet based on Western dietary habits, which could not well demonstrate the role of Chinese dietary patterns in the association between air pollution and hypertension. Moreover, we have a wide variety of foods collected from the population and there are strong correlations between intakes of various foods and nutrients, making it difficult to study their effects on disease. Reduced rank regression analysis (RRR) is a statistical method for reduce the dimension that can be a good solution to this problem. The response variables can be selected based on prior experience, while the food group under investigation serves as an independent variable. It identifies the optimal linear combination of food groups that effectively accounts for the variability in selected response variables, such as nutrients and disease-related biomarkers. This approach, initially proposed by Hoffman and widely adopted in nutritional epidemiology, combines both prior and posterior methods [11]. It determines dietary patterns by identifying the appropriate mediator between diet and disease, thereby addressing the limitations of priori methods that may lack significant correlation with the target disease and posteriori methods that cannot be generalized to the population [12, 13]. In this study, the RRR method was employed to analyze dietary patterns, which not only maximized the explanation of hypertension-related response variables but also reflects the actual dietary status of Jiangsu residents. As a result, we obtained insights into the association between specific dietary patterns and air pollutants with hypertension.

## Materials and methods

### Study design and population

**Fig. 1:**
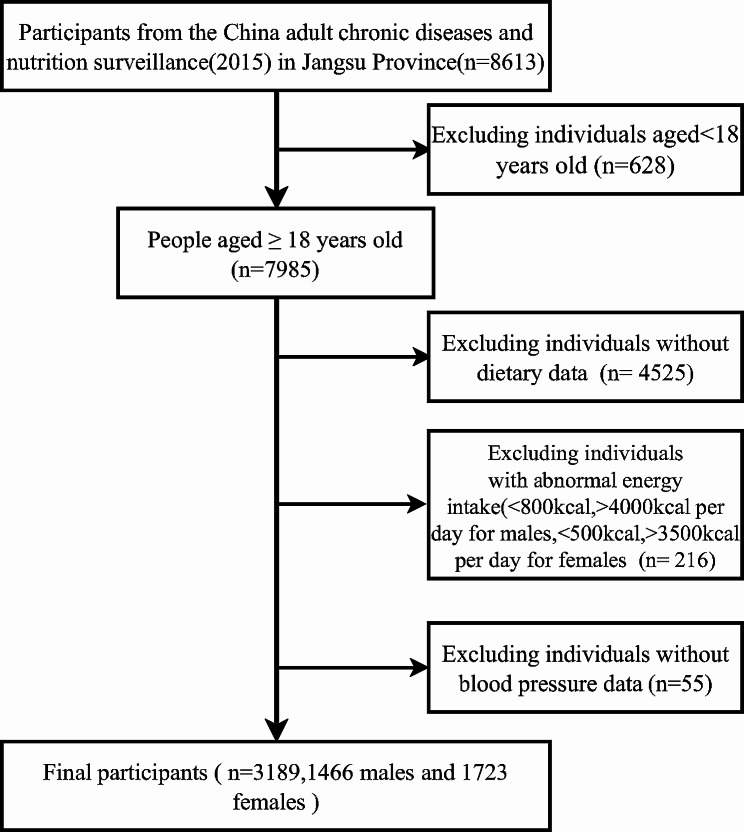
Flowchart for Inclusion and Exclusion of Study Population

This study was conducted based on the 2015 survey of chronic diseases and nutrition surveillance among Chinese adults in Jiangsu Province. It incorporated stratified factors such as urban and rural areas, regions, and working conditions by selecting monitoring counties from Chinese mortality surveillance system. A total of 60 streets, villages, and towns across 13 counties and districts were chosen as monitoring sites in the province using a multi-stage cluster random sampling method to select individuals who had resided there for at least 6 months within the year prior to the survey. The survey encompassed inquiries, anthropometric measurements, laboratory tests, and dietary surveys. Individuals with incomplete dietary data, missing outcome variables, or abnormal energy intake (< 800kcal, > 4000 kcal per day for males and < 500kcal, > 3500 kcal per day for females) were excluded, left a total of 3189 subjects in this study. (Fig. 1.)

### Air Pollution exposure Assessment

Exposure estimates of PM_2.5_ and O_3_ were obtained from the Tracking Air Pollution in China (TAP) platform (http://tapdata.org.cn). The TAP 10 km PM_2.5_ model was developed based on a two-stage machine learning approach, incorporating various datasets including PM_2.5_ observation data, satellite remote sensing Aerosol Optical Depth (AOD) data, Community Multiscale Air Quality (CMAQ) simulation outputs, meteorological reanalysis data, land use information, altitude measurements and population statistics. The two-stage model employed decision tree methodology to establish the relationship between missing satellite data and other parameters in order to bridge the gap. The PM_2.5_ data with a resolution of 1 km was retrieved with a machine learning model on the basis of a 10 km resolution data set by fusing high-resolution satellite remote sensing AOD data and environmental space data such as road network, the detailed method was shown in the following article [14–17]. O_3_ data was a spatio-temporal continuous grid of the maximum daily 8-hour average concentration of ozone [18–20]. The model for estimating PM_2.5_ and O_3_ concentrations in this study performed satisfactorily, with a cross-validation R^2^ of 0.80–0.88 and root-mean-squared error (RMSE) of 13.9–22.1 µg/m^3^ in the PM_2.5_ exposure estimate model. The estimates are highly correlated with the in situ observations of the maximum daily 8 h averaged O_3_ (R^2^ = 0.70) and the mean modeling error is 26 µg/m^3^ [17, 18]. The average concentration of PM_2.5_ and O_3_ in the three years preceding the survey year (2015) was utilized as the pollutant concentration based on geocoding residents’ home addresses in this study [20].

### Dietary assessment

Dietary data were obtained by 24-hour dietary recall questionnaire and food frequency questionnaire (FFQ) through face-to-face interviews. The types and quantities of food except for oil, salt and other condiments were recorded for three consecutive days. A separate questionnaire was employed to inquire about participants’ consumption information regarding household condiments and cooking oil over the past month, the weighing method was employed to investigate the consumption of oil and seasonings among all members of the household. The individual consumption of condiments and cooking oils was determined by considering the average consumption within a household and accounting for the size of its population. The food frequency questionnaire inquired about the average consumption and frequency of various foods consumed over a 12-month period. It encompassed 69 food groups, including the staple food, beans and products, vegetable, fungi and algae, fruit, dairy products, etc. To accurately estimate nutrient intake for each subcategory in the food frequency questionnaire, we utilized data from the dietary recall method to determine the composition of individual foods within each subcategory. This information was then combined with the China food composition Tables (2002, 2018) to calculate the corresponding nutrient intake based on their respective proportions [21, 22].

### Outcome measurement and definition

The blood pressure was measured in a quiet and comfortable environment, ensuring that the survey participants adhered to the specified measurement conditions. They refrained from engaging in vigorous physical activity for at least one hour prior to the measurement, abstained from smoking for 30 min beforehand, remained relaxed throughout the process, and refrained from consuming any caffeinated beverages such as coffee or tea. Omron HBP1300 electronic sphygmomanometer was consistently utilized in the monitoring points. Each subject was measured three times, with an interval of one minute between each measurement. The coincidence rate of the two blood pressure monitoring results should exceed 95%, and the mean of the three results was taken as the blood pressure value. Hypertension is defined as a systolic blood pressure (SBP) of ≥ 140mmHg and/or a diastolic blood pressure of ≥ 90mmHg [23]. Participants’ blood was collected early in the morning (8–12 h fasting). Then, the blood was centrifuged and fasting blood glucose (FBG) was measured using the glucose oxidase method. High blood glucose was defined as: FBG ≥ 7.0 mmol/L. According to the established criterion(Joint Committee for Developing Chinese Guidelines on Prevention and Treatment of Dyslipidemia in Adults, 2007): Hypercholesterolemia was defined as total cholesterol(TC) ≥ 6.22 mmol/L, hypertriglyceridemia was defined as triglycerides (TG) ≥ 2.26 mmol/L, hypoalphalipoproteinemia was defined as high-density lipoprotein cholesterol (HDL-C) < 1.04 mmol/L, and hyperbetalipoproteinemia was defined as low-density lipoprotein cholesterol(LDL-C) ≥ 4.14 mmol/L, participants with one or more abnormities of these lipid levels above were defined as having dyslipidemia [24]. The Cardiovascular disease (CVD) included heart disease and stroke. It was defined by medical diagnosis as reported to the following questionnaire item: ‘Have you been diagnosed with heart attack, coronary heart disease, angina, congestive heart failure, other heart problems, or stroke by a doctor?’ [25].

### Dietary patterns analysis

Dietary patterns were derived using RRR, seven response variables, namely dietary fiber, thiamine, riboflavin, vitamin C, calcium, magnesium, and potassium were selected due to their demonstrated inverse association with hypertension [13, 26]. Based on the nutritional composition and intake of various foods, a total of 25 food groups were identified as predictive variables: rice and its products, wheat flour products, grains, potatoes, beans and their products, fried foods, fresh vegetables, dried vegetables, pickled vegetables, milk and dairy products, pork, poultry meat (including chicken), beef (including cattle), sheep and other livestock meat (excluding poultry), animal offal (such as liver), seafood (including fish and shellfish), eggs and egg products, peanuts and other nut seeds, fungi and algae, fruits, carbonated drinks, coffee, other sugary drinks, fresh vegetable juice, alcohol and snacks. Seven factor loadings and their corresponding dietary patterns were obtained, and only the first dietary pattern was retained for subsequent analysis due to its superior explanatory power in relation to all response variables. The dietary score of the survey subjects was then calculated using this first factor loading as a metric [27].

### Covariates assessment

Covariates were determined based on a synthesis of previously published literature on the association between pollutants and hypertension. Age group(< 45 years old, 45–65 years old, > 65 years old), body mass index (BMI)(< 24 kg/m^2^, 24–28 kg/m^2^, > 28 kg/m^2^), education level (primary school or below, junior middle school, high school and above), ethnicity (Han/other), marital status (divorced/widowed/unmarried/separate (married couple), married/cohabiting), family history of hypertension, the categorization of occupations was based on three levels that correspond to the degree of manual labor involved, while smoking status was classified into current smoking, former smoking, and never smoking categories based on frequency, drink (yes or no), annual household income in accordance with the extent of three digits into three levels, physical activity was categorized into three levels based on the weekly metabolic equivalent (MET), measured in minutes per week, the main source of cooking fuel were divided into solid fuels (firewood/charcoal/wood, coal), clean fuels(gas/liquefied petroleum gas/natural gas/biogas, solar energy/electricity) and other fuels.

### Statistical analysis

The basic information of the population includes continuous variables expressed as mean ± standard deviation and categorical variables expressed as numbers and percentages. The long-term effects of PM_2.5_ and O_3_ on hypertension were assessed separately from different sex using multivariable logistic regression analysis. The MICE package of R software was used to fill in the missing values of annual household income. The above variables were expressed as the mean and standard deviation for continuous variables and the rate for categorical variables when describing the baseline characteristics. The model was augmented with a multiplicative interaction term between PM_2.5_ and dietary score to explore potential diet-related factors, while the likelihood ratio test was employed to assess the presence of a multiplicative interaction between these two variables. The dietary pattern score was categorized into three quantile levels, and the association between long-term PM_2.5_ exposure and hypertension was evaluated at each level. Statistical significance was determined using a two-tailed P-value threshold of less than 0.05. The RRR dietary analysis was conducted using Stata15(Stata Crop LLC), and all other analyses were performed by R (version 4.2.3).

## Results

### Descriptive statistic

Finally, 3189 participants were included for analysis. The overall prevalence of hypertension was 38.7%. The systolic and diastolic blood pressures exhibited a slight elevation in males compared to females(*P* < 0.001), alongside a higher prevalence of hypertension among males(44.0%) as opposed to females(34.1%). The distribution of PM_2.5_ and O_3_ exposure levels, annual household income level, the main source of cooking fuel exposure, marital status, and family history of hypertension did not exhibit significant sex differences. However, males exhibited higher levels of educational attainment, tobacco consumption, and alcohol consumption compared to females(*P* < 0.001) (Table 1).

**Table 1:** Sex-stratified baseline characteristics of participants

Variables	Male (*N* = 1466)	Female (*N* = 1723)	P value
SBP (Mean ± SD)	137.5 ± 17.9	133.3 ± 20.1	< 0.001
DBP (Mean ± SD)	81.9 ± 10.7	77.8 ± 10.3	< 0.001
Age (Mean ± SD)	56.5 ± 14.5	54.3 ± 14.7	< 0.001
BMI (Mean ± SD)	25.0 ± 3.3	24.8 ± 3.6	0.225
Dietary score (%)			< 0.001
Q1[-4.8, -1.1]	405 (27.6%)	658 (38.2%)	
Q2[-1.1, 0.6]	492 (33.6%)	571 (33.1%)	
Q3[0.6,16.1]	569 (38.8%)	494 (28.7%)	
PM_2.5_, µg/m^3^(%)			0.711
Q1[53.7,67.9]	525 (35.8%)	593 (34.4%)	
Q2[67.9,72.0]	458 (31.2%)	552(32.0%)	
Q3[72.0,88.1]	483 (32.9%)	578(33.5%)	
O3, µg/m^3^(%)			0.769
Q1[121.4,133.1]	506 (34.5%)	613(35.6%)	
Q2[133.1,142.0]	518(35.3%)	590 (34.2%)	
Q3[142.0,154.2]	442 (30.2%)	520 (30.2%)	
Hypertension (%)			< 0.001
Yes	645(44.0%)	588 (34.1%)	
No	821(56.0%)	1135 (65.9%)	
Annual household Income, yuan (%)			0.922
Q1[1200,40000]	508 (34.7%)	605 (35.1%)	
Q2[40000,72000]	495(33.8%)	585 (34.0%)	
Q3[72000,1440000]	463 (31.6%)	533 (30.9%)	
Physical activity (%)			0.002
Low	535 (36.5%)	528 (30.6%)	
Moderate	479 (32.7%)	595 (34.5%)	
High	452 (30.8%)	600 (34.8%)	
The main source of cooking fuel (%)			0.690
Solid fuel ^a^	238 (16.2%)	299 (17.4%)	
Clean fuel ^b^	1225 (83.6%)	1421 (82.5%)	
Other fuels ^c^	3 (0.2%)	3 (0.2%)	
Ethnicity (%)			1.000
Han	1460 (99.6%)	1716 (99.6%)	
Other	6 (0.4%)	7 (0.4%)	
Age (years)			< 0.001
< 45	305 (20.8%)	441 (25.6%)	
45–65	685 (46.7%)	839 (48.7%)	
≥ 65	476 (32.5%)	443 (25.7%)	
BMI (kg/m^2^)			< 0.001
< 24	565 (38.5%)	764 (44.3%)	
24–28	655 (44.7%)	650 (37.7%)	
≥ 28	246 (16.8%)	309 (17.9%)	
Family history of hypertension (%)			0.323
Yes	649 (44.3%)	794 (46.1%)	
No	817 (55.7%)	929 (53.9%)	
Education qualifications (%)			< 0.001
Primary schools and below	480 (32.7%)	819 (47.5%)	
Junior middle school	505 (34.4%)	483 (28%)	
High school and above	481 (32.8%)	421 (24.4%)	
Marital status (%)			0.269
Divorced/Widowed/Unmarried/separate (married couple)	87 (5.9%)	120 (7.0%)	
Married/Cohabiting	1379 (94.1%)	1603 (93.0%)	
Occupation (%)			< 0.001
low intensity	813 (55.5%)	1114 (64.7%)	
moderate intensity	127 (8.7%)	69 (4.0%)	
high intensity	314 (21.4%)	377 (21.9%)	
other	212 (14.5%)	163 (9.5%)	
Smoking (%)			< 0.001
Current smoker	752 (51.3%)	32 (1.9%)	
Previous smoker	237 (16.2%)	12 (0.7%)	
Never	477 (32.5%)	1679 (97.4%)	
Drinking (%)			< 0.001
Yes	926 (63.2%)	311 (18%)	
No	540 (36.8%)	1412 (82%)	
Total energy (Mean ± SD)	2172.18 ± 678.7	1797.1 ± 595.6	< 0.001
Diabetes mellitus (%)			0.217
Yes	149 (10.2%)	152 (8.9%)	
No	1309 (89.8%)	1562 (91.1%)	
Dyslipidemia (%)			< 0.001
Yes	517 (37.5%)	471 (28.8%)	
No	860 (62.5%)	1166 (71.2%)	
Cardiovascular disease (%)			0.305
Yes	193 (13.2%)	205 (11.9%)	
No	1273 (86.8%)	1518 (88.1%)	

SD standard deviation, SBP systolic blood pressure, DBP diastolic blood pressure, BMI Body Mass Index. Q1、Q2、Q3 are tri-sectional quantiles

^a^ Including firewood/charcoal/wood, coal

^b^ Including gas/liquefied petroleum gas/natural gas/biogas, solar energy/electricity

^c^ Fuels excluding solid and clean fuels

### Reduced rank regression factor score

**Fig. 2:**
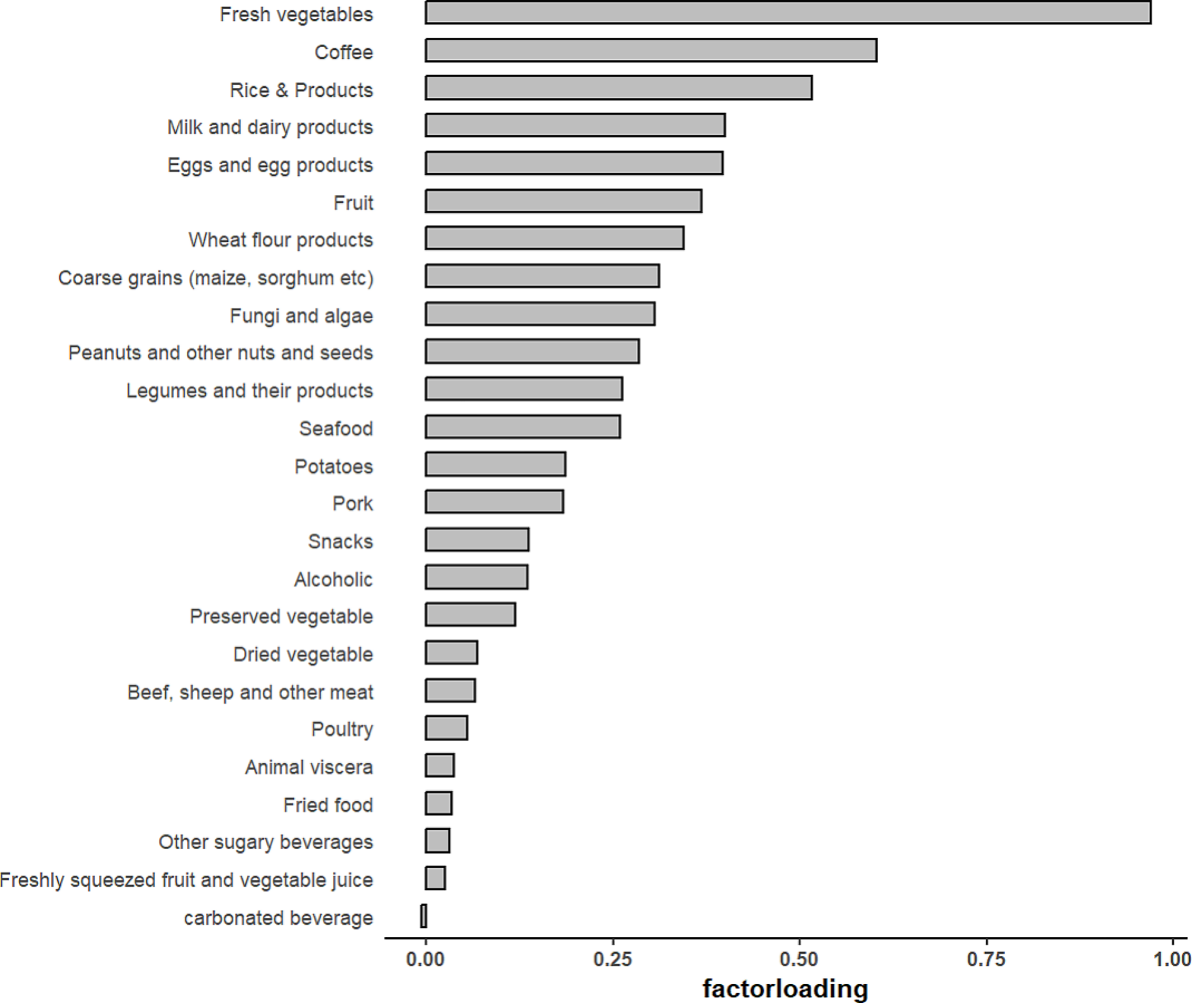
Reduced-rank regression analyses of factor loading plot for each food group

Figure 2 presented factor loads for all food groups, characterizing dietary patterns using foods with absolute factor loads exceeding 0.25. The first dietary pattern accounted for 60.26% of the response variable and explained 8.17% of the variance in food consumption, primarily characterized by a substantial intake of fresh vegetables, coffee, rice and related products, dairy products, eggs and egg products, fungi and algae, fruits, wheat flour products, whole grains, legumes and their derivatives as well as peanuts and other nut seeds, and was designated as the traditional southern dietary pattern [28].

### Association of dietary patterns, air pollutants and hypertension based on multivariate logistic regression

#### Association between 3-year average concentrations of PM_2.5_ and O_3_ and hypertension

In the single pollution model of PM_2.5_ and O_3_, after adjusting for confounding factors such as age and BMI, it was revealed that males exhibit higher susceptibility to long-term exposure to PM_2.5_, while no significant association was observed with O_3_ exposure. Long-term exposure to PM_2.5_ demonstrated a positive correlation with a 42% increased risk in the highest quintile compared to the lowest quintile of PM_2.5_ exposure (95%CI:1.08–1.87). Conversely, females displayed higher sensitivity towards long-term exposure to O_3_, with no significant risk associated with PM_2.5_ exposure; however, long-term exposure to O_3_ exhibited a positive correlation with hypertension risk infemales, where the risk of hypertension in the highest quantile of O_3_ concentration was 1.61 times higher than that in the lowest quantile (95%CI: 1.15, 2.23). (Table 2.)

**Table 2:** Association between the average concentrations of PM_2.5_ and O_3_ and hypertension

Air pollution	Male			Female		
	Crude Model	Adjust model1	Adjust model2	Crude model	Adjust model1	Adjust model2
PM_2.5_(µg/m^3^)						
Q1[53.7,67.9]	Reference	Reference	Reference	Reference	Reference	Reference
Q2[67.9,72.0] Q3[72.0,88.1]	1.13(0.88–1.46) 1.46 (1.14–1.87)	1.16 (0.89–1.52) 1.48 (1.13–1.92)	1.12(0.85–1.48) 1.42(1.08–1.87)	0.92 (0.72–1.17) 0.82 (0.64–1.05)	1.00 (0.75–1.32) 0.77 (0.58–1.02)	1.00(0.75–1.33) 0.77(0.58–1.03)
O_3_(µg/m^3^)						
Q1[121.4,133.1] Q2[133.1,142.0] Q3[142.0,154.2]	Reference 0.98 (0.77–1.26) 1.15 (0.89–1.49)	Reference 1.06 (0.78–1.43) 1.20 (0.86–1.68)	Reference 1.04(0.77–1.41) 1.18(0.85–1.64)	Reference 0.69 (0.54–0.88) 0.98 (0.77–1.25)	Reference 0.93 (0.69–1.26) 1.55 (1.13–2.14)	Reference 0.95(0.70–1.29) 1.61(1.15–2.23)

Crude Model was the 95% CI unadjusted for confounders;

Adjust model1 was the 95% CI derived by controlling for age, BMI, annual household income, the main source of cooking fuel, physical activity level, family history of hypertension, education level, marital status, occupation, smoking habits and alcohol consumption

Adjust model2 was the 95% CI derived by controlling for age, BMI, annual household income, the main source of cooking fuel, physical activity level, family history of hypertension, education level, marital status, occupation, smoking habits, alcohol consumption, diabetes mellitus, dyslipidemia and cardiovascular disease

#### Association between RRR dietary pattern score and hypertension

Table 3 presented the association between dietary pattern scores obtained through RRR and hypertension in both males andfemales. We observed a significant inverse correlation between the highest digit of the dietary score and the risk of hypertension in males(OR = 0.73, 95% CI: 0.56, 1.00, P_trend_<0.05), indicating that the RRR-derived dietary pattern exerted a protective effect against hypertension in this group. Conversely, no significant correlation was found among females(OR = 0.99, 95% CI: 0.74, 1.34).

**Table 3:** Association between the dietary pattern score of RRR and hypertension

Dietary score	Male		Female	
	OR (95%CI)	P trend	OR (95%CI)	P trend
Q1 Q2 Q3	Reference 0.93(0.70–1.25) 0.73(0.56-1.00)	0.009	Reference 0.92(0.70–1.22) 0.99(0.74–1.34)	0.819

CI, confidence interval. OR, Odds Ratio

95% CI for the association between dietary scores and hypertension in males and females were adjusted by controlling for age, BMI, annual household income, the main source of cooking fuel, physical activity level, family history of hypertension, education level, marital status, occupation, smoking habits, alcohol consumption, diabetes mellitus, dyslipidemia and cardiovascular disease

#### Association of RRR dietary pattern score with air pollution and hypertension in males

**Fig. 3:**
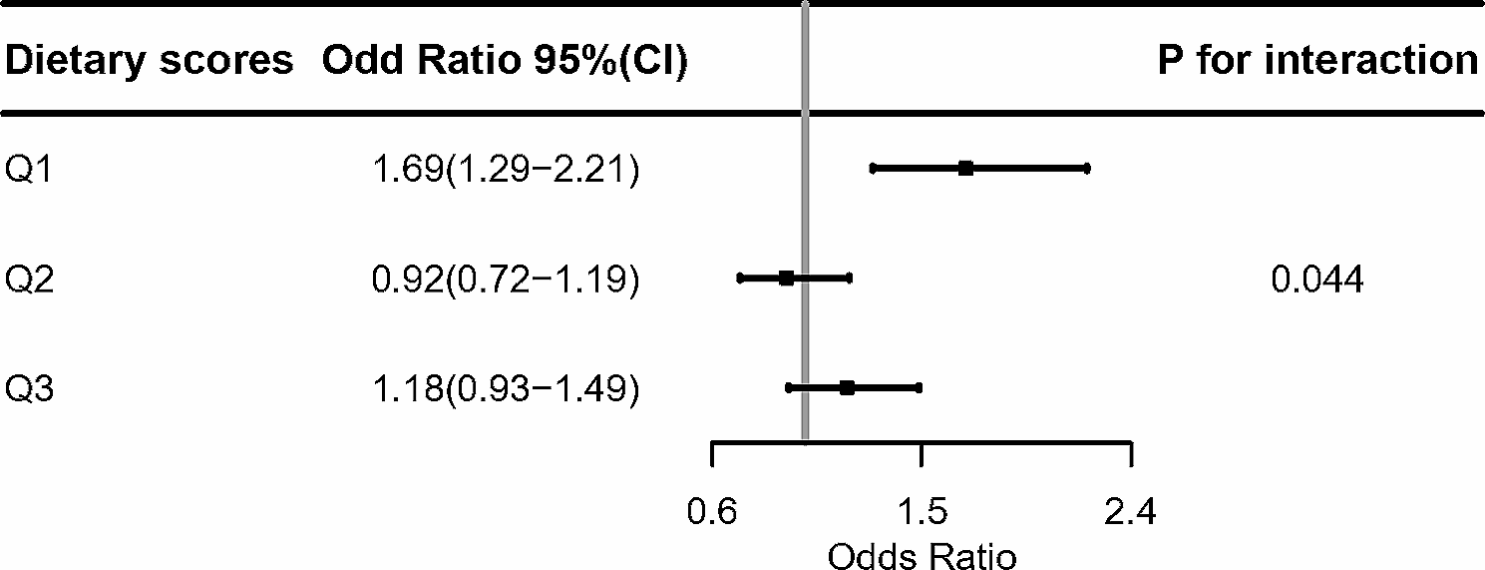
The dietary pattern, PM_2.5_ and risk of hypertension among males in Jangsu Province

Figure 3 showed the results stratified by categories of dietary pattern scores. There was an interaction between dietary scores and PM2.5 exposure in males with hypertension (*P* for interaction = 0.046). Among males with the lowest tertile of dietary scores, the OR for hypertension was 1.69(95%CI: 1.29, 2.21). In the two higher quantiles of dietary score, the risk of hypertension in males was 0.92 (95%CI: 0.72,1.19) and 1.18(95%CI:0.93,1.49), respectively, there was no significant statistical difference between dietary scores and PM_2.5_ exposure. However, the P-trend was 0.012, and the risk of hypertension among the three groups was statistically different.

Dietary score Q1, lowest quartile of the dietary score tertiles. Dietary score Q2, dietary score tertiles 2nd quartile. Dietary score Q3, highest quartile of the dietary score tertiles.

Figure 3 showed the association between PM_2.5_ exposure and the risk of hypertension at three levels of dietary pattern scores. *P* for interaction was calculated by adding the cross-product term of the dietary pattern score and concentration of PM_2.5_ in the multivariable logistic model. OR, Odds Ratio; CI, confidence interval.

## Discussion

In this study, dietary patterns associated with hypertension were constructed by using reduced rank regression analysis to explore whether dietary patterns could affect the association of long-term PM_2.5_ and O_3_ exposure on hypertension in adult males and females. Our findings revealed both the susceptibility of adult malesand femalesto long-term exposure to PM_2.5_ and O_3_, as well as identified specific dietary patterns that could mitigate the risk of particulate pollution-induced hypertension.

The multivariate logistic regression analysis revealed a significant association between high PM_2.5_ exposure and an increased risk of hypertension in males, while no such association was observed in females. Previous studies have demonstrated a positive correlation between long-term exposure to PM_2.5_ and the incidence of hypertension, with a progressively stronger association observed as the duration of PM_2.5_ exposure increases [29–31]. While the majority of the findings were representative of the entire population, emerging evidence suggested potential sex disparities in the relationship between PM_2.5_ and hypertension. The incidence of hypertension or diabetes was not significantly associated with long-term PM_2.5_ exposure in a prospective cohort study of black females, even after controlling for temporal and spatial variations in PM_2.5_ [32]. Xie et al. investigated the long-term impact of fine particulate matter on hypertension in a large cohort of 39,348,119 Chinese individuals aged 20 to 49. They found that the population-attributable risk of PM_2.5_ exceeding the threshold (47.9 µg/m^3^) was estimated at 2.3% (95% CI, 2.2 to 2.4). Notably, malesexhibited a higher susceptibility to long-term exposure to PM_2.5_ with an attributable fraction of hypertension reaching 3.6% (95% CI, 3.4 to 3.7), compared to only 0.5% (95% CI, 0.3 to 0.6) in females [33]. A separate cross-sectional study involving nearly 1 million Chinese patients investigated the correlation between long-term exposure to PM_2.5_ and hypertension, revealing that advanced age, male, and obesity exhibit heightened susceptibility to PM_2.5_ exposure consistent with our findings [34]. However, Curto et al.'s study investigating the association between PM_2.5_ and hypertension among adult males and females in 28 urban areas of India revealed that males exhibited lower susceptibility to PM_2.5_, while females showed a positive correlation between exposure to PM_2.5_ and hypertension. This discrepancy may be attributed to differences in residential patterns within the study population, with local females spending approximately 83% of their time at home compared to only 57% for males [35]. Longitudinal studies conducted by Adar et al. did not find any significant association between hypertension and long-term exposure to air pollutants in adults, possibly due to the relatively low average annual PM_2.5_ exposure of 17 µg/m^3^, which contrasted with the higher level of 69.6 µg/m^3^ observed in our study [36]. Simultaneously, we observed a statistically significant disparity in the association between long-term O_3_ exposure and hypertension risk among females(OR = 1.55, 95% CI: 1.13, 2.14), while no correlation was found among males(OR = 1.20, 95% CI: 0.86, 1.68). Consistent with our results, another time-series analysis exploring O_3_ and CVD mortality in Jiangsu province exhibited a higher susceptibility to O_3_ compared to me [37]. In the analysis of the relationship between six air pollutants and the incidence rate of high blood pressure, it was found that particles PM_2.5_ and PM10 have a stronger association withmales, while gaseous pollutants SO_2_ and O_3_ have a stronger impact on females [38]. Naturally, there existed certain studies whose findings were incongruous with our own [39, 40].

The sex-specific nature of the association of air pollutants with hypertension may be related to physical fitness, social functioning, and sex-related biological differences between malesand females [41]. Sex-related biological factors, such as lung volume, airway hyperresponsiveness, and systemic conditioning by hormones, along with differences in smoking prevalence between sex, variations in the accuracy of exposure allocation based on residence, and disparities in exposure to indoor allergens and cleaning agents may contribute to observed differences [42]. In general, the differences were mainly caused by exposure patterns and biological responses. Although the precise mechanism underlying the association between air pollutants and hypertension remained elusive, one frequently proposed hypothesis was that air pollutants can elicit a range of inflammatory factors (such as cytokines, activated immune cells, platelets, etc.) to enter the systemic circulation. This process subsequently induced systemic inflammation and oxidative stress, leading to systemic endothelial dysfunction, autonomic imbalance, and progression of atherosclerosis [38, 43]. According to previous epidemiological studies, long-term exposure to PM2.5 had a greater impact on males, possibly due to their higher frequency and density of outdoor activities that expose them to high concentrations of particulate matter [44]. It also has been suggested that air pollution and cigarette smoking may affect cardiovascular health through the same pathways [45]. Therefore, additional exposure to smoking may enhance the effect of PM_2.5_ on hypertension incidence, which may also partially explain the greater effects of PM_2.5_ in males, as the smoking prevalence is much higher in Chinese males than females (54.5% vs. 3.7%, *P* < 0.001) [46]. Lastly, a fraction of the total effect of ambient PM on cardiovascular mortality may be mediated through sustained long-term effects of air pollution on atherosclerosis. Whereas premenopausal females are protected against atherosclerosis by endogenous hormones [47].Regarding the sensitivity of females to chronic ozone exposure compared to males, oxidative and inflammatory effects of smoking may dominate to such an extent that the additional exposure to O_3_ may not further enhance effects along the same pathways [48]. On the other hand, O_3_ is formed through sunlight-driven reactions between volatile organic compounds and nitrogen oxides. High concentrations of ozone are often accompanied by high temperatures, which can have a more pronounced effect on the body. The possible sex difference in fitness conditions, vascular characteristics, percentage of body fat, and hormone levels may be responsible for the greater difficulty in dissipating heat for females. In addition, compared with males, females have slightly greater airway reactivity, it is possible that dose–response relations may be detected more easily in females than in males [49].

Epidemiological evidence suggested that modifying one’s diet or increasing the intake of antioxidant dietary supplements can effectively reduce the disease burden caused by air pollutants [50]. However, limited research has been conducted on the impact of dietary patterns on the susceptibility to air pollution-induced hypertension. The only two studies conducted in China have demonstrated that adherence to the DASH diet pattern can mitigate the risk of hypertension associated with exposure to PM_2.5_, while the Mediterranean diet also exhibited a potential for reducing such risk; however, its effect was comparatively less significant than that of the DASH diet [51, 52]. The logistic regression model of hypertension in this study revealed a significant interaction between the traditional southern pattern and PM_2.5_ exposure concentration, indicating that the risk of hypertension exhibited a decreasing trend with increasing quantiles of dietary score. Specifically, this dietary pattern was found to potentially mitigate long-term PM_2.5_ exposure and reduce the risk of hypertension among males, while no such association was observed among females. The traditional southern pattern determined through RRR exhibited similarities to those identified in previous studies conducted in South China [53]. Notably, a protective effect against hypertension was observed in various food groups including vegetables, coffee, rice and its products, dairy products, eggs and egg products, bacteria and algae, fruits, wheat flour products, whole grains, legumes and their derivatives, nuts and seeds such as peanuts, as well as seafood. Fresh vegetables and fruits were rich sources of potassium, which effectively mitigated lipid peroxidation. Additionally, they served as excellent reservoirs of antioxidant vitamins C and E [54]. The primary constituent of coffee was caffeine, and despite its acute hypertensive effect, regular consumers of coffee can develop tolerance to the stimulatory effects of caffeine [55]. In addition, the coffee contained soluble fiber, polyphenols, and potassium, which may confer a protective effect against hypertension [56]. The recent cohort study demonstrated that mushroom and algal polysaccharides exhibited various physiological activities, including immunomodulatory, antioxidant, and anti-inflammatory effects. These activities have been found to contribute to a reduction in all-cause mortality among elderly individuals in China [57].

The research on sex differences in the association between diet and hypertension was relatively limited, with a majority of studies focusing on males. However, it was crucial to consider the metabolic, hormonal, and microbial variations between males and females as these dietary disparities undoubtedly influenced disease outcomes [58]. The traditional southern pattern obtained in this study demonstrated a sex-specific protective effect against hypertension, with a significant association observed in malesbut not in females. Related studies have shown that the Mediterranean diet was more strongly associated with males. For younger adults, glucose and insulin levels were more stable in males than in females who adhered to the Mediterranean diet, and the cardiovascular protective effect of the Mediterranean diet was more obvious in males than in females [59, 60]. Similarly, in the study conducted by ADAMS et al., the vegetarian diet exhibited a significant association with decreased risk of cardiovascular disease among male participants, while no such correlation was observed among female participants [61]. In South Korea’s genome and epidemiological study, no significant sex differences were observed in the association between a plant-based diet and hypertension. However, an unhealthy plant-based diet was found to be linked to hypertension, especially among females. No significant associations between other dietary patterns and hypertension were observed for both sexes. Notably, a diet rich in whole grains and legumes showed an inverse association with hypertension specifically in Korean females but not males [62, 63]. The sex disparity in the relationship between diet and hypertension may be attributed to divergent responses of males and females to stimulation of the renin-angiotensin system, such as the reduction in estrogen levels that triggers activation of the renin-angiotensin system and subsequent elevation of angiotensin II, thereby leading to an increase in blood pressure [64]. Further investigation is warranted to explore additional sex-specific disparities in the relationship between dietary patterns and hypertension.

This study employed a reduced rank regression approach in constructing a dietary pattern, aiming to investigate the potential of Jiangsu residents’ diet in mitigating hypertension risk caused by air pollutants. This analysis effectively captured the intricate interplay among dietary habits, air pollutants, and hypertension among Jiangsu residents, providing valuable evidence on the role of diet in mediating this association. Admittedly, this study also had certain limitations. Firstly, it was a cross-sectional study and thus unable to establish a causal relationship between exposure and outcome. Secondly, the utilization of the food frequency method might introduce recall bias in dietary data collection. Moreover, our reliance on air pollutant data from the nearest monitoring station based on participants’ home addresses restricted our ability to track individual exposure dynamics accurately and reflected population-wide pollutant exposure comprehensively. Additionally, we did not examine the impact of concurrent exposure to PM_2.5_ and O_3_. Our analysis only investigated the association between individual exposure to pollution and hypertension. Lastly, no specific association was observed between traditional southern pattern and air pollutants or hypertension in males; therefore, further evidence is warranted through larger sample sizes.

## Conclusion

In this cross-sectional study, our findings demonstrated that adherence to a traditional southern pattern was associated with a reduced risk of long-term PM_2.5_-induced hypertension inmales. Furthermore, we observed sex-specific differences in the sensitivity to both dietary patterns and air pollutants. Our evidence underscored the importance of adopting healthy dietary patterns and considering sex disparities in air pollution for effective prevention and control of hypertension. Further investigations are warranted to validate our results.

## Data Availability

The datasets used and/or analyzed during the current study are available from the corresponding author on reasonable request.

## Abbreviations

AOD: Aerosol Optical Depth
BMI: Body mass index
CMAQ: Community Multiscale Air Quality
CVD: Cardiovascular disease
DASH: Dietary Approaches to Stop Hypertension
DALYs: Disability adjusted life years
FBG: Fasting blood glucose
FFQ: Food frequency questionnaire
HDL-C: High-density lipoprotein cholesterol
LDL-C: Low-density lipoprotein cholesterol
MET: Metabolic equivalent
OR: Odds ratio
PM: Particulate matter
RRR: Reduced rank regression
SD: Standard deviation
TC: Total cholesterol
TG: Triglycerides
